# Supplementary material for: Arabidopsis SEC13B Interacts with Suppressor of Frigida 4 to Repress Flowering
Source: Int J Mol Sci. 2023 Dec 8;24(24):17248. doi: 10.3390/ijms242417248 (PMC10744139; doi:10.3390/ijms242417248)
Supplement: Supplementary file 1 [file ijms-24-17248-s001.zip › Supplemental Figure.pdf]

```
                 WD40 domain
SEC13A 1    MPGQKIETGHEDIVHDVQMDYYGKRIATASSDCTIKITGVSNNGGSQQLATLTGHRGPVW  60
SEC13B 1    MPPQKIETGHSDTIHDVVMDYYGKRVATASSDCTIKITGVSNSGGSQHLATLTGHRGPVW  60

              WD40 domain                         WD40 domain
SEC13A 61   EVAWAHPKYGSILASCSYDGQVILWKEGNQNQWTQDHVFTDHKSSVNSIAWAPHDIGLSL  120
SEC13B 61   QVAWAHPKFGSLLASCSYDGQIILWKEGNQNQWTQAHVFTDHKVSVNSIAWAPHELGLSL  120

                                          WD40 domain
SEC13A 121  ACGSSDGNISVFTARADGGWDTSRIDQAHPVGVTSVSWAPATAPGALVSSGLLDPVYKLA  180
SEC13B 121  ACGASDGNISVFSARADGGWDTTKIDQAHPVGVTSVSWAPATEPGALVSSGMIDPVYKLA  180

                                         WD40 domain
SEC13A 181  SGGCDNTVKVWKLANGSWKMDCFPALQKHTDWVRDVAWAPNLGLPKSTIASGSQDGKVII  240
SEC13B 181  SGGCDSTVKVWKFSNGSWKMDCFPALNKHTDWVRDVAWAPNLGLPKSTIASGSEDGKVII  240

                              WD40 domain
SEC13A 241  WTVGKEGEQWEGKVLKDFMTPVWRVSWSLTGNLLAVSDGNNNVTVWKEAVDGEWEQVTAV  300
SEC13B 241  WTIGKEGEQWEGTVLKDFKTPVWRVSWSLTGNLLAVSDGNNNVTVWKESVDGEWEQVTVV  300

SEC13A 301  EP
SEC13B 301  EP
```

Figure S1. The protein sequence of SEC13A showed 89% identity and 93.7% similarity with that of SEC13B. Identical amino acids are highlighted in black; Substitutable amino acids are highlighted in gray; Heterotactic amino acids are in white.

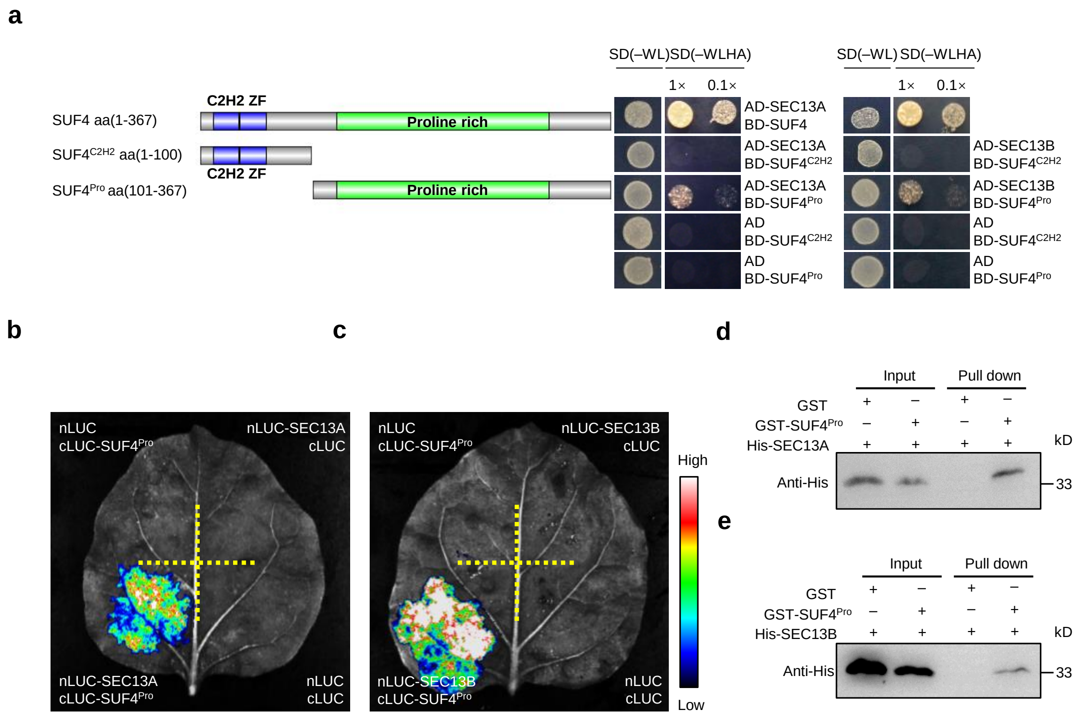

Figure S2. SUF4 interacts with SEC13A and SEC13B via the Pro domain. (a) SEC13A and SEC13B interacts with SUF4^{Pro} in yeast. Schematic represented the truncated fragments of SUF4. C2H2, C2H2 zinc finger; Pro, Proline rich domain. (b, c) LCI assays show that SEC13A and SEC13B interacts with SUF4^{Pro} in *N. benthamiana.* (d, e) Expressed SEC13A and SEC13B interact with SUF4^{Pro} in vitro. All protein samples were immunoprecipitated with anti-GST beads and immunoblotted with anti-His antibodies. The symbols "+"and "–" represent the presence and absence of the corresponding proteins.

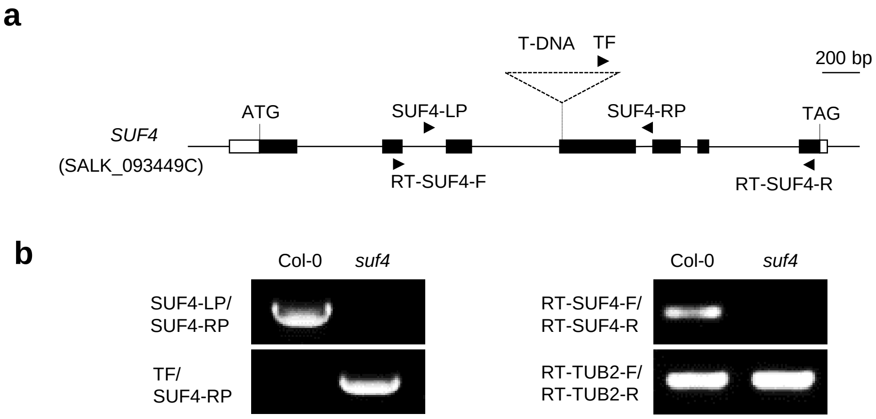

Figure S3. Identification of *suf4* mutant. (**a**) Gene structure of *SUF4*. The filled white box indicates UTRs, black box indicates exon, lines between the boxes indicate introns, and dashed triangles indicate insertional T-DNAs. Black arrows indicate the positions and directions of the primers. SUF4-LP, SUF4-RP and TF were used for identification of *suf4* mutant (SALK_093449C); RT-SUF4-F/R and RT-TUB2-F/R were used for RT-PCR. (**b**) Genotyping of Col and mutant by PCR analysis, and transcript levels of *SUF4* in Col and mutant determined by RT-PCR.

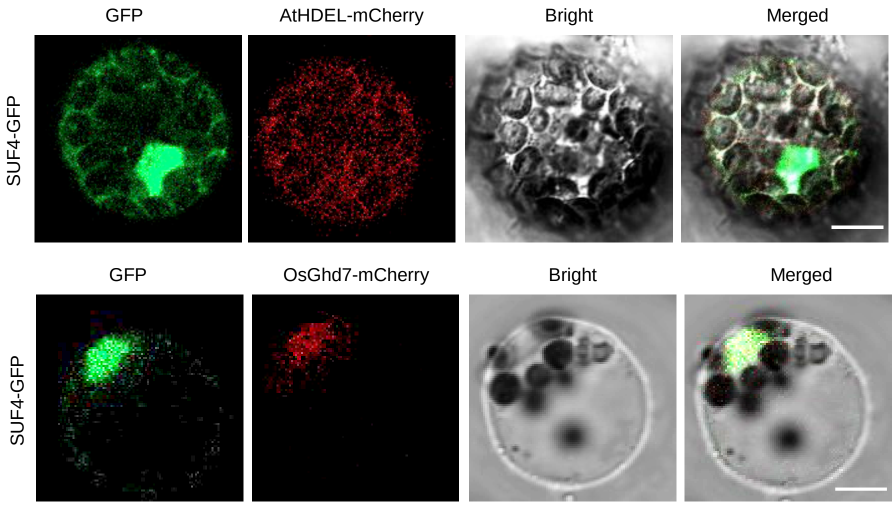

Figure S4. SUF4 is localized to ER and nucleus. SUF4-GFP was co-transformed into *Arabidopsis* protoplasts with AtHDEL-mCherry and OsGhd7-mCherry, respectively. AtHDEL-mCherry was used as ER marker. OsGhd7-mCherry was used as nuclear marker. The images were taken in green (GFP fluorescence), red (mCherry fluorescence), bright and red-green combination (merged) channels, respectively. Bar = 20 μm.

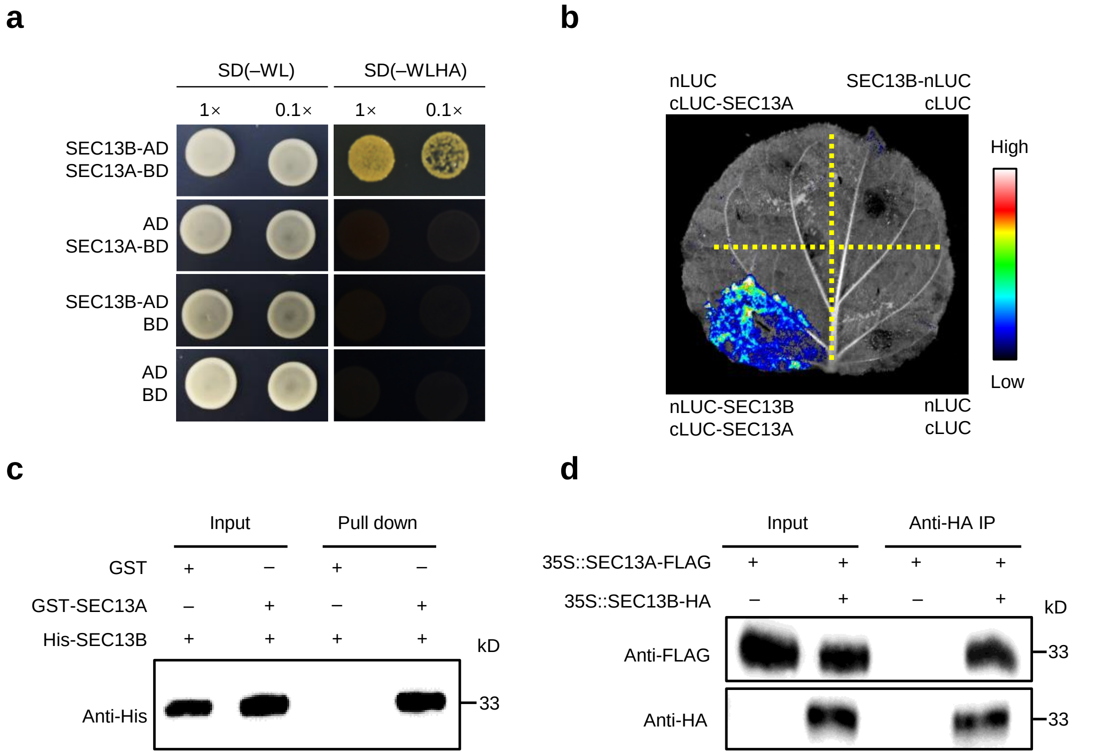

Figure S5. SEC13A interacts with SEC13B. (**a**) SEC13A interacts SEC13B in yeast. (**b**) LCI assay showed that SEC13A interacts with SEC13B in *N. benthamiana.* (**c**) Expressed SEC13A interacts with SEC13B in vitro. All protein samples were immunoprecipitated with anti-GST beads and immunoblotted with anti-His antibodies. (**d**) Co-IP assays validate that SEC13A interacts with SEC13B in *N. benthamiana*. The symbols "+" and "–" represent the presence and absence of the corresponding proteins.
